# Computational Design and Optimization of Peptide Inhibitors for SIRT2

**DOI:** 10.3390/ph17091120

**Published:** 2024-08-24

**Authors:** Heba A. Alkhatabi, Fatmah M. A. Naemi, Reem Alsolami, Hisham N. Alatyb

**Affiliations:** 1 Department of Medical Laboratory Sciences, Faculty of Applied Medical Science, King Abdulaziz University, Jeddah 21589, Saudi Arabia; halkhattabi@kau.edu.sa (H.A.A.); abuahmed440775@gmail.com (R.A.); 2 Hematology Research Unit (HRU), King Fahd Medical Research Center (KFMRC), King Abdulaziz University, Jeddah 21589, Saudi Arabia; 3 Center of Artificial Intelligence for Precision Medicine (CAIPM), King Abdulaziz University, Jeddah 21589, Saudi Arabia; 4 Histocompatibility and Immunogenetics Laboratory, Prince Abdulmajed Dialysis Center, King Fahd General Hospital, Ministry of Health, Jeddah 21589, Saudi Arabia; fnaemi@moh.gov.sa; 5 Department of Biochemistry, Faculty of Science, King Abdulaziz University, Jeddah 21589, Saudi Arabia

**Keywords:** SIRT2 inhibitors, cyclic peptides, molecular dynamics simulation, computational biology, binding free energy calculations

## Abstract

Sirtuin 2 (SIRT2), an NAD+-dependent deacetylase, is crucial for regulating vital physiological processes, including aging, DNA repair, and cell cycle progression. Its abnormal activity is linked to diseases such as Parkinson’s disease, cancer, and metabolic disorders, making it a potential target for therapeutic intervention. While small molecule inhibitors have been studied, peptide-based inhibitors offer a promising alternative due to their selectivity and bioavailability. This study explores the effects of converting the naturally occurring cyclic inhibitor peptide of SIRT2 (S2iL5) into a non-cyclic form by replacing a residue with FAK (LYS + CF3CO^−^). The new peptide sequence, Tyr-His-Thr-Tyr-His-Val-FAK (LYS)-Arg-Arg-Thr-Asn-Tyr-Tyr-Cys, was modeled to confirm its stable conformation. Docking studies and MM/GBSA calculations showed that the non-cyclic peptide had a better binding free energy (−50.66 kcal/mol) compared to the cyclic S2iL5 (−49.44 kcal/mol). Further mutations generated 160,000 unique peptides, screened using a machine learning-based QSAR model. Three promising peptides (Peptide 1: YGGNNVKRRTNYYC, Peptide 2: YMGEWVKRRTNYYC, and Peptide 3: YGGNGVKRRTNYYC) were selected and further modeled. Molecular dynamics (MD) analyses demonstrated that Peptide 1 and Peptide 2 had significant potential as SIRT2 inhibitors, showing moderate stability and some structural flexibility. Their best binding free energies were −59.07 kcal/mol and −46.01 kcal/mol, respectively. The study aimed to enhance peptide flexibility and binding affinity, suggesting that optimized peptide-based inhibitors can interact effectively with SIRT2. However, further experimental validation is necessary to confirm these computational predictions and evaluate the therapeutic potential of the identified peptides.

## 1. Introduction

Sirtuin 2 (SIRT2), an NAD+-dependent deacetylase that is present in the central nervous system (CNS) and serves as a lysine deacetylase and defatty-acylase, has been associated with neurological disorders [1,2]. Conserved regulators of aging, sirtuin proteins have lately come to be recognized as significant moderators of a number of diseases that often manifest later in life, including cancer, diabetes, cardiovascular disease, and neurodegenerative diseases [3]. Aberrant activity of SIRT2 has been associated with neurodegenerative diseases, particularly Parkinson’s disease [4]. SIRT2 is implicated in the regulation of alpha-synuclein, a protein that aggregates abnormally in Parkinson’s disease [5]. SIRT2 has been linked to various types of cancer [6]. Its role in cell cycle regulation and apoptosis suggests that dysregulation of SIRT2 activity may contribute to tumorigenesis. SIRT2 is involved in metabolic regulation, and its dysfunction is associated with metabolic disorders, including obesity and diabetes [7].

While SIRT2 is widely recognized for its role in promoting the progression of neurological disorders, it also serves to safeguard the brain under specific conditions [1]. Of the seven known mammalian sirtuin isoforms (SIRT1–7), SIRT2, which is mostly cytosolic, is one of the least understood [8,9]. This includes the nuclear proteins SIRT6 and SIRT7, mitochondrial proteins SIRT3, SIRT4, and SIRT5, and both the nucleus and the cytoplasm include SIRT1 and SIRT2 [10]. Both increased SIRT1 and decreased SIRT2 reduce neurodegeneration, which may raise cautionary flags for the creation of widely acting sirtuin-based medications for these kinds of diseases [11]. Sirtuins influence repair pathways by altering chromatin accessibility or deacetylating repair factors [12]. Inducing cell death requires the silencing of both SIRT1 and SIRT2, emphasizing the critical nature of inactivating both proteins in order to achieve cytotoxic effects [13]. SIRT2 modulates the expression of genes involved in lipid metabolism, which is vital for maintaining liver integrity and regulating lipid metabolic homeostasis [14].

Several small-molecule inhibitors are found for the direct inhibition of SIRT2. AGK2, SirReal2, Tenovin-6, and TM are sirtuin inhibitors that have been designed to selectively inhibit SIRT2 [15]. Similarly, inhibition of SIRT2 by AEM1 and AEM2 compounds causes increased activation of p53 by decreasing SIRT2-dependent p53 deacetylation. The most potent inhibitor of SIRT2 (6,8-dibromo-2-pentylchroman-4-one (I)) was identified through in vitro and in silico analysis, including pharmacophore screening and potency evaluation for potential therapeutic applications [16]. SIRT2 is identified as a potential therapeutic target for neurodegenerative disease [17].

Compared to medium-sized inhibitors like peptides, small molecules may struggle to provide the necessary structural complexity and stability required for effective binding to protein surfaces [18]. Peptide-based inhibitors offer a novel approach that may overcome some limitations of small molecules, such as specificity and bioavailability [19]. Many other small-molecule inhibitors are found, but they lack peptide-based inhibitors. Thus, this study identifies a peptide-based inhibitor for SIRT2 using an advanced computational approach and machine learning algorithms.

Peptide-based inhibitors offer a novel approach that may overcome some limitations of small molecules, such as specificity and bioavailability. This study employed machine learning models to generate non-cyclic peptides, which were subsequently screened using the Quantitative Structure–Activity Relationship (QSAR). In addition, the stability and flexibility of the peptides were confirmed using molecular docking and molecular dynamics simulation. Additionally, the technique of MM/GBSA was employed to determine the binding free energy and assess the strength of the peptide–protein interaction with SIRT2. Ultimately, a novel inhibitor peptide was found that exhibited superior efficacy compared to the previously identified inhibitor of SIRT2.

## 2. Results and Discussion

### 2.1. Peptide Modeling and Docking

S2iL5 forms a binding interaction with the active site of SIRT2 in the PDB structure. The peptide’s macrocyclic structure enables it to assume a conformation that precisely fits into the enzyme’s binding pocket. The trifluoroacetyllysine moiety binds to certain residues in the active site, hence increasing the inhibition of SIRT2’s deacetylase activity. S2iL5 has demonstrated superior efficacy in inhibiting SIRT2 activity compared to numerous other known inhibitors. The enhanced effectiveness of this substance is ascribed to its distinct structural characteristics, such as the presence of a large cyclic ring and the inclusion of a trifluoroacetyllysine residue. These traits contribute to a greater ability to bind to and specifically target SIRT2.

**Figure 1:**
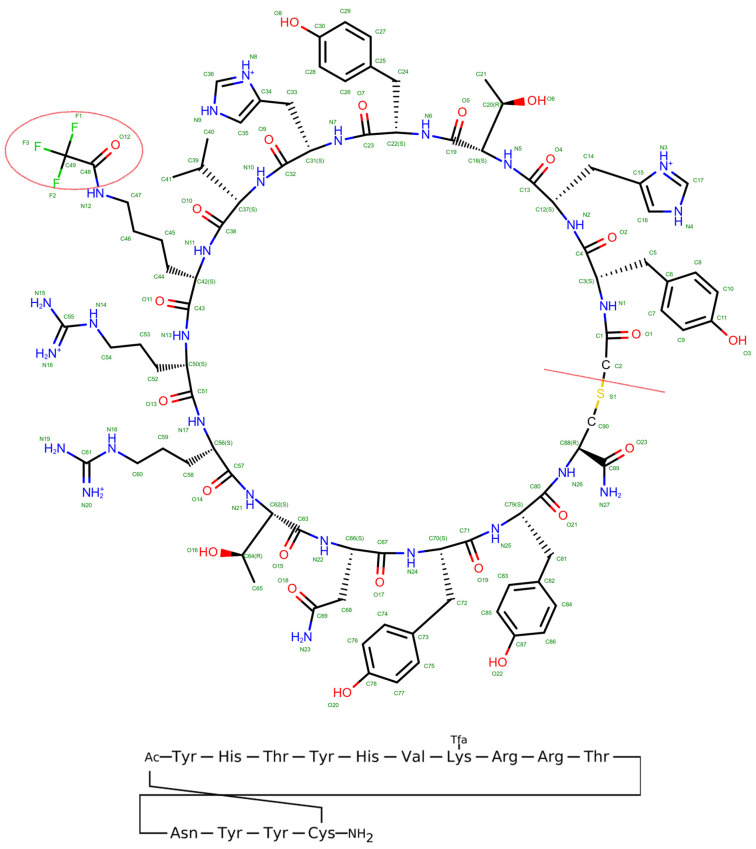
Molecular structure of the native cyclic peptide S2iL5. Key functional groups and side chains of amino acids are depicted, emphasizing the peptide’s spatial conformation and potential interaction sites.

**Figure 2:**
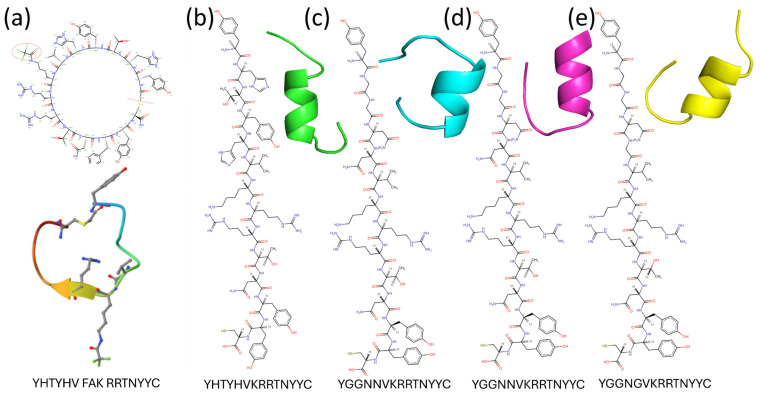
Structural and molecular representations of various peptides with different sequences. Each panel illustrates the chemical structure and 3D conformation of the peptides, highlighting differences in folding patterns and secondary structures: (**a**) cyclic peptide S2iL5, (**b**) non-cyclic peptide, (**c**) peptide 1, (**d**) peptide 2, (**e**) peptide 3.

As shown in Figure 1, the native peptide sequence Acy-Tyr-His-Thr-Tyr-His-Val-FAK-Arg-Arg-Thr-Asn-Tyr-Tyr-Cys-NH_2_ was modified to exclude the peptide linker and the modified residue, FAK (LYS + CF_3_CO), resulting in the new non-cyclic peptide sequence Tyr-His-Thr-Tyr-His-Val-LYS)-Arg-Arg-Thr-Asn-Tyr-Tyr-Cys. The native cyclic structure is dependent on the presence of the peptide linker and the modified residue, which facilitates the cyclization process. When these elements are excluded, the peptide can no longer form a closed ring structure, hence converting it into a linear or non-cyclic peptide form. This linear form retains the amino acid sequence but lacks the specific conformational constraints and properties imparted by cyclization. This modification aimed to investigate the impact of FAK on the peptide’s structure and interaction with the target protein, potentially enhancing stability or binding affinity. Figure 2 shows five different peptides, each characterized by distinct sequences and structures. The peptides, labeled with sequences YHTYHVFAKRRTNYYC, YHTYHVKRRTNYYC, YGGNNVKRRTNYYC, YGGNVKRRTNYYC, and YGCGNVKRRTNYYC are depicted in both 2D chemical structures and 3D conformations. While the peptide in Figure 2a displays a cyclic structure, suggesting increased stability or specific activity due to its constrained loop, the peptides in Figure 2b through Figure 2e demonstrate helical conformations, which are indicative of structural stability and potential biological functions. The presence of cysteine residues in some sequences points to possible disulfide bonds, enhancing the peptides’ rigidity. These structural characteristics hint at the peptides’ potential applications in drug development, structural biology, and interaction studies, where specific sequences and conformations may offer targeted therapeutic or functional benefits.

**Figure 3:**
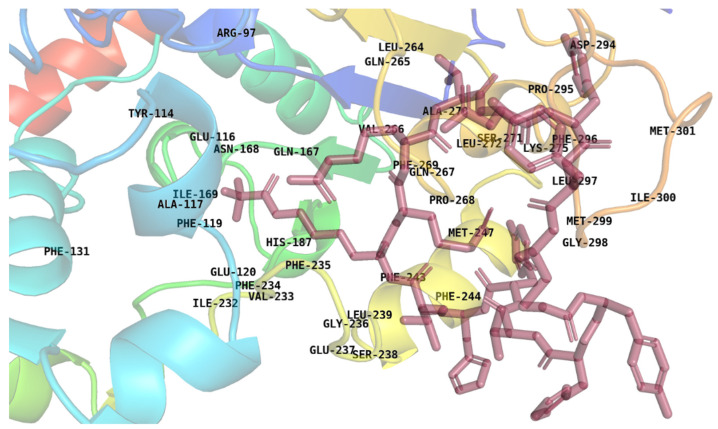
The binding residues were identified using the PyMOL tool, the binding residues are those located within a 6 Å radius around the known inhibitor (S2iL5) in the crystal structure SIRT2.

The modified peptide, excluding the ε-trifluoroacetyllysine, was subjected to structure prediction using PEP-FOLD3. This tool employs a de novo approach to generate plausible three-dimensional structures of peptides, leveraging the structure prediction algorithm for accurate modeling. The predicted structure of the modified peptide was docked onto the SIRT2 protein using the H-Dock server. The residues PHE131, TYR114, ARG97, GLU116, ASN168, ILE169, ALA117, PHE119, PHE244, PHE243, MET247, PRO268, PHE269, ILE300, LEU300, LEU272, SER271, GLU120, PHE234, VAL233, ILE232, HIS187, PHE235, GLN167, LEU239, GLY236, GLU237, SER238, GLN267, VAL266, MET301, ALA270, ARG97, LEU264, GLN265, PHE269, GLN267, PRO268, MET247, PHE243, PHE244, GLY298, MET299, LEU297, LYS275, PHE296, PRO295, ASP294, MET301, ILE300, MET299, and GLY298 are the binding site residues around the known inhibitor that are used for docking, as shown in Figure 3. In Figure 3, the brown color stick model shows the cyclic peptide S2iL5, and the protein SIRT2 is shown in rainbow color (Figure S1). A total of 100 docking models were generated to explore the possible binding modes of the peptide to the protein.

### 2.2. MMGBSA for the Docked Complex of New Peptide

The evaluation of the altered non-cyclic peptide’s binding interactions with SIRT2 encompassed a thorough process that incorporated docking investigations, estimations of binding free energy, and molecular dynamics (MD) simulations. All 100 docked models were utilized for subsequent binding free energy research. Subsequently, molecular dynamics simulations were performed to achieve energy minimization for all changed peptide variations, aiming to achieve ideal conformations and avoid any steric conflicts. The calculation of binding free energy was used to rank the models as shown in Supplementary Table S1. Model number 54, which exhibited the highest binding free energy of −37.19 kcal/mol, was selected for a 100 ns molecular dynamics (MD) simulation. The control, S2iL5, was also included in the simulation for comparative analysis. This methodology ensured a comprehensive evaluation of the peptide’s ability to inhibit SIRT2, identifying model 54 as a highly promising option for further investigation.

### 2.3. MD of Native Peptide and Modified Peptide (100 ns)

**Figure 4:**
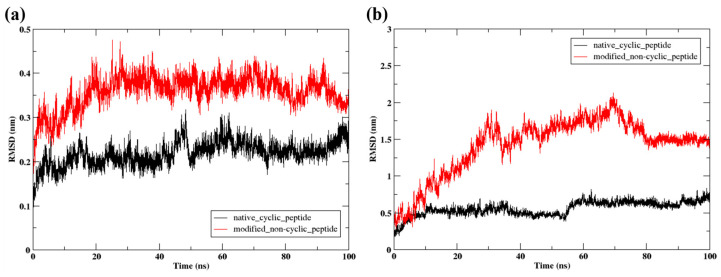
(**a**) RMSD of the protein Cα atoms of the protein and (**b**) RMSD of the peptides during the 100 ns MD simulation.

The RMSD of the protein is shown in Figure 4a. The protein, which complexes with native cyclic peptide, showed stable confirmation up to 50 ns at 0.2 nm. At 50 ns, it slightly deviated from 0.2 nm to 0.28 nm, and afterward, it showed a stable configuration at 0.2 nm. However, the protein complex with the modified cyclic peptide initially exhibited fluctuations at a distance of 0.3 nm, which subsequently increased to higher fluctuations at 0.4 nm. Eventually, the complex reached a state of stability. This suggested the RMSD for the modified non-cyclic peptide increases and then stabilizes at a higher value than the native cyclic peptide, indicating more significant structural deviations. Moreover, the RMSD of the peptide is shown in Figure 4b. Here, at the initial stage, the native cyclic peptide showed stability at 0.5 nm up to 55 ns, and after 55 ns, it showed slight fluctuation at 0.55 nm, then showed stability during the run. Nevertheless, the modified non-cyclic peptide exhibited a highly unstable and minimal variation ranging from 0.5 nm to 1.8 nm within the first 25 ns. Subsequently, over the time interval from 25 ns to 70 ns, the value fluctuated within the range of 1.5 nm to 2 nm. Finally, in the last 20 ns, it reached a steady state at 1.5 m. Overall, it suggested that the native cyclic peptide is more structurally stable over time compared to the modified non-cyclic peptide, as evidenced by its consistently lower RMSD values in both RMSD.

### 2.4. MM/GBSA

**Figure 5:**
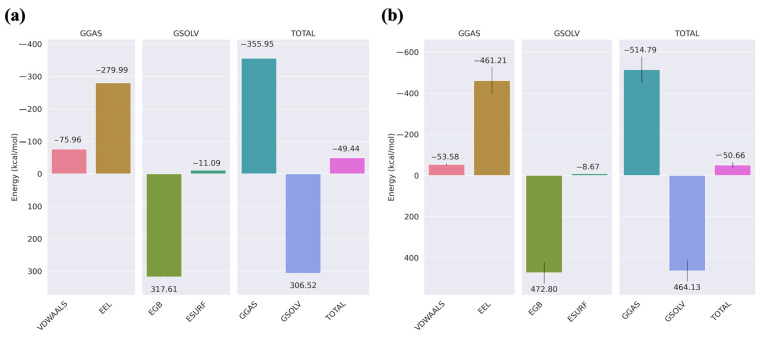
MMGBSA of last 20 ns: (**a**) native cyclic peptide, (**b**) modified non-cyclic peptide.

The binding free energy of the protein–peptide complex was determined in Figure 5. Here, the total binding energy of the native cyclic peptide complex was −49.44 kcal/mol, which is the combination of GGAS and GSOLV, and the total binding energy of the modified non-cyclic peptide complex was −50.66 kcal/mol with the combination of GGAS and GSOLV. When evaluating the binding interactions and stability of peptides, the non-cyclic peptide emerges as potentially superior despite the small difference in total binding energy. The van der Waals interactions (−53.58 kcal/mol for the non-cyclic peptide and −75.95 kcal/mol for the cyclic peptide) suggest that the non-cyclic form offers greater flexibility, allowing it to adapt more readily to different binding environments. This adaptability can be crucial in dynamic biological systems where flexibility enhances molecular recognition. Furthermore, the significant electrostatic energy difference (−181.22 kcal/mol) indicates that the non-cyclic peptide provides more favorable charge–charge interactions, enhancing specificity and binding strength through precise complementary positioning. The differences in generalized Born electrostatic solvation energy (EGB) imply that the non-cyclic peptide interacts more effectively with the solvent, stabilizing polar regions and potentially improving solvation dynamics. Despite the total binding energy difference being only 1.22 kcal/mol, the non-cyclic peptide’s superior charge distribution, flexibility, and solvation dynamics may contribute to enhanced binding affinity and specificity, making it a more promising candidate for applications where adaptability and precise molecular interactions are vital. This showed that the non-cyclic peptide complex showed the strongest binding affinity. Therefore, the modified non-cyclic peptide was selected for further analysis.

### 2.5. Energy Decomposition and Interaction

**Figure 6:**
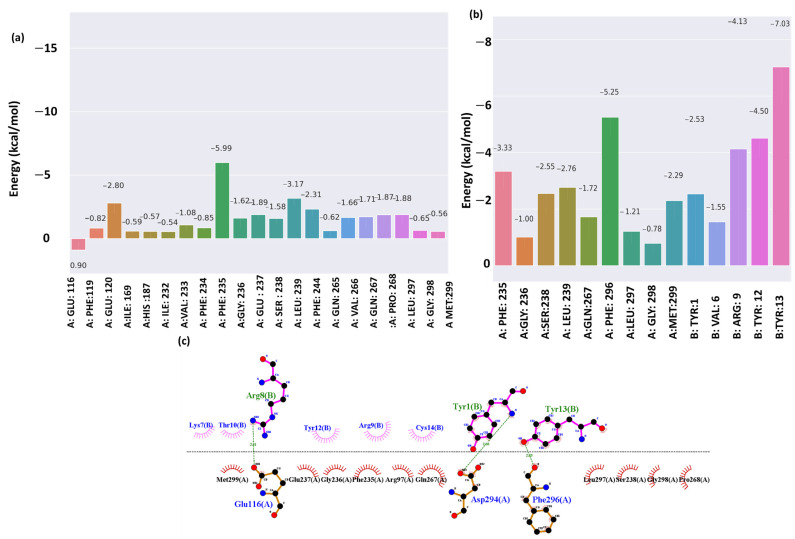
Energy Decomposition: (**a**) Cyclic peptide (S2Li5), (**b**) non-cyclic peptide, and (**c**) Interaction of the final complex of modified non-cyclic peptide–protein in a 100 ns trajectory.

Figure 6a shows the energy decomposition of the non-cyclic peptide S2Li5. The residue Phe236 contributes significantly to the stability with an energy of −5.99 kcal/mol, indicating strong interactions, possibly through hydrogen bonds or hydrophobic forces. Other residues, like A:Phe:244 and A:Phe:119, also play important stabilizing roles with energies of −3.17 kcal/mol and −2.80 kcal/mol, likely due to aromatic interactions. In contrast, A:Glu:116 has a positive energy contribution of 0.90 kcal/mol, suggesting a potentially destabilizing effect, possibly from electrostatic repulsion or steric hindrance. Figure 6b,c shows the energy decomposition and interactions of the modified non-cyclic peptide. Here, residue Tyr13 showed −7.03 kcal/mol decomposition energy; this residue has the most negative binding energy, indicating a strong interaction, which corresponds to the multiple hydrogen bonds it forms in the interaction diagram. This highlights its crucial role in stabilizing the complex. Another residue Tyr12 showed −4.50 kcal/mol, aligning with its significant pi-stacking interactions in the diagram. Aromatic interactions are known for their contribution to binding stability. Arg9 shows moderate binding energies (−2.53 and −1.55 kcal/mol, respectively). The interaction diagram suggests that Arg8 (close to Arg9) may participate in electrostatic interactions, which, while not as strong as hydrogen bonds or pi-stacking, still contribute to the binding. Overall, the energy decomposition graph and interaction analysis offer a complete overview of how specific residues in the B chain contribute to the overall binding energy through different molecular interactions. Notably, Tyr13(B) and Tyr12(B) play a significant role, with Tyr13(B) contributing through hydrogen bonds and Tyr12(B) through pi-stacking. Additionally, Arg8(B) and the region around it contribute through electrostatic interactions, although these have a lesser impact compared to the previously mentioned interactions.

### 2.6. Combination Mutation, QSAR, and Clustering

The residues of the non-cyclic modified peptide that did not show any interactions were modified. Further, mutations were performed in these positions. The original peptide sequence was “YHTYHVKRRTNYYC” by targeting mutations at positions 2, 3, 4, and 5. The selection of positions 2, 3, 4, and 5 for single mutations was based on their lack of significant interactions with the protein. These residues are crucial for maintaining the peptide’s secondary structure and stability, allowing them to be altered without compromising the peptide’s integrity. While positions 6, 7, and 11 showed van der Waals interactions, as indicated in Figure 6c, the focus remained on positions 2, 3, 4, and 5 because of their specific roles in preserving the peptide’s overall conformation. By targeting these positions, the mutations were designed to explore functional changes without disrupting the peptide’s essential structural properties. All possible single-residue mutations were produced using the 20 standard amino acids at the specified positions with Python’s itertools product, resulting in 160,000 unique peptide variants. Combining all amino acid possibilities, and substituting them into the original sequence, these variants were constructed.

Furthermore, the Quantitative structure–activity relationship (QSAR) model was developed to predict protein–ligand interactions. Data of 1049 protein–peptide complexes was taken from the PDBbind database for training and modeling QSAR. After cleaning the data, 876 complexes were used further for training. The protein and peptide sequences were encoded using multiple techniques before being split into training, validation, and testing sets. Various models, including Random Forest, Ridge Regression, Gradient Boosting, and XGBoost, were trained and optimized with hyperparameter tuning via Optuna to minimize the root mean square error (RMSE). An Isolation Forest was employed to detect and remove outliers, enhancing the robustness of the models.

**Figure 7:**
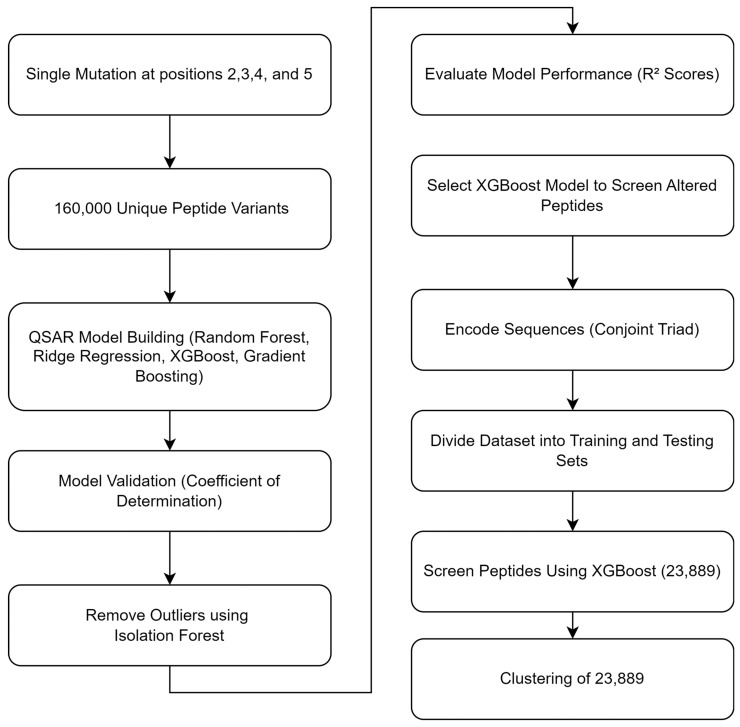
Flowchart illustrating the process of combination mutation, QSAR model building, validation, and clustering of peptide variants.

The models’ performances were evaluated using R^2^ scores, and the final model was saved using pickle. R^2^, or the coefficient of determination, is a statistical measure that represents the proportion of the variance in the dependent variable that is predictable from the independent variables in a regression model. It is a key metric used to assess the goodness-of-fit of a model According to the data presented in Table 1, the XGBoost model demonstrated the highest R^2^ value (0.995 for the training set and 0.696 for the test set), indicating superior performance. Therefore, it was selected to screen the altered peptides. In order to predict activity, a total of 160,000 distinct peptide variants were obtained and prepared. These variants included a target protein sequence (SIRT2) and the associated altered peptides. The sequences were encoded using the Conjoint Triad technique and labeled for prediction. The dataset was divided into separate training and testing sets. In post-screening using this XGBoost model, 23,889 mutant peptides had superior predictive activity in comparison to the non-cyclic control peptide, S2iL5. A flow chart regarding the mutation and QSAR is given in Figure 7.

### 2.7. Clustering

**Figure 8:**
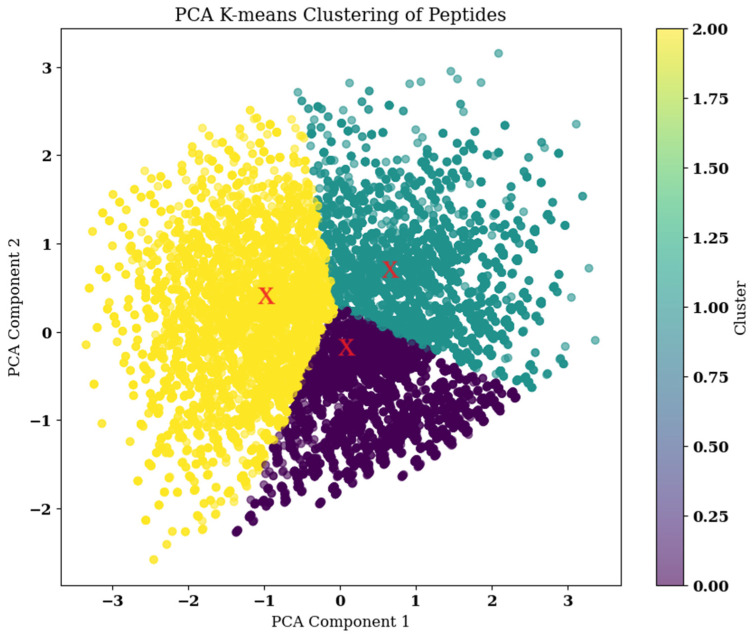
K-means Clustering with Centroids. Each point represents a compound, and the colors denote the three identified clusters. The red “X” markers indicate the centroids of the respective clusters, which are the mean positions of the data points within each cluster. The centroids serve as representative points for the clusters.

Further, clustering of the 23,889 mutant peptides was performed. Hydrophobicity values and molecular weights were calculated for each peptide using predefined scales. Subsequently, the data were normalized using standard scaling. Principal Component Analysis (PCA) was then applied to reduce the dimensionality of the feature space to two components. Silhouette analysis was performed using a range for k″ (or clusters) from 2 to 10. For each k, the silhouette score is calculated, which identifies the ideal number of clusters (or k) to perform k-means clustering. The silhouette score, along with the number of clusters, is given in Supplementary Table S2. The optimal number of clusters shown by the silhouette analysis is three, so the number of clusters required for the analysis is three. K-means clustering is performed on the reduced data from PCA, with the number of clusters set to three. The cluster assignments and centroids are visualized on a scatter plot, where each point represents a peptide, and the centroids are marked with red crosses. The peptides that are closest to each centroid are determined by calculating their Euclidean distances to the centroids in the reduced space as shown in Figure 8. Three peptides that are nearest to each centroid are gathered for additional investigation. These closest peptides were Peptide 1: YGGNNVKRRTNYYC, Peptide 2: YMGEWVKRRTNYYC, and Peptide 3: YGGNGVKRRTNYYC.

### 2.8. Peptide Modeling and Docking

PEPFOLD.3 was used for peptide modeling of the three peptides: Peptide 1: YGGNNVKRRTNYYC, Peptide 2: YMGEWVKRRTNYYC, Peptide 3: YGGNGVKRRTNYYC, and the top models were selected for further analysis. Target docking was performed using the H-Dock server for the top three peptides using the same protocol as used for the modified non-cyclic peptide. One hundred models retrieved from docking were further used for an MD simulation run till the energy minimization and free binding energy were calculated using MMGBSA. It was found that the best models were model number 16 for Peptide 1 (−37.62 kcal/mol), model number 30 for Peptide 2 (−40.93 kcal/mol), and model number 65 for Peptide 3 (−32.45 kcal/mol), which had the highest free binding energy. These three models were selected for further MD simulation of 300 ns. The ADME properties of these three peptides were identified using the ProtParam tool, which is given in Table 2. The comparison of the three peptides reveals distinct differences in their molecular characteristics, despite sharing the same number of amino acids (14). Peptide 2 has the highest molecular weight (1869.15) and a slightly lower theoretical pI (9.11) compared to Peptide 1 and Peptide 3 (both with a pI of 9.63). The presence of a negatively charged residue in Peptide 2 may contribute to its lower pI. The instability index categorizes all three peptides as unstable, with Peptide 2 being the most unstable (78.71) and Peptide 3 being the least (55.46). Despite differences in atomic composition, all three peptides share an identical aliphatic index (20.71), suggesting similar aliphatic content and structural characteristics. However, the grand average of the hydropathicity (GRAVY) scores varies, with Peptide 2 being the least hydrophilic (−1.229), indicating slightly higher hydrophobicity, while Peptide 1 is the most hydrophilic (−1.579). Overall, the variations in molecular weight, pI, and GRAVY scores suggest differences in solubility and stability that could impact their biological functions and interactions.

### 2.9. Molecular Dynamics Simulation

#### 2.9.1. RMSD

**Figure 9:**
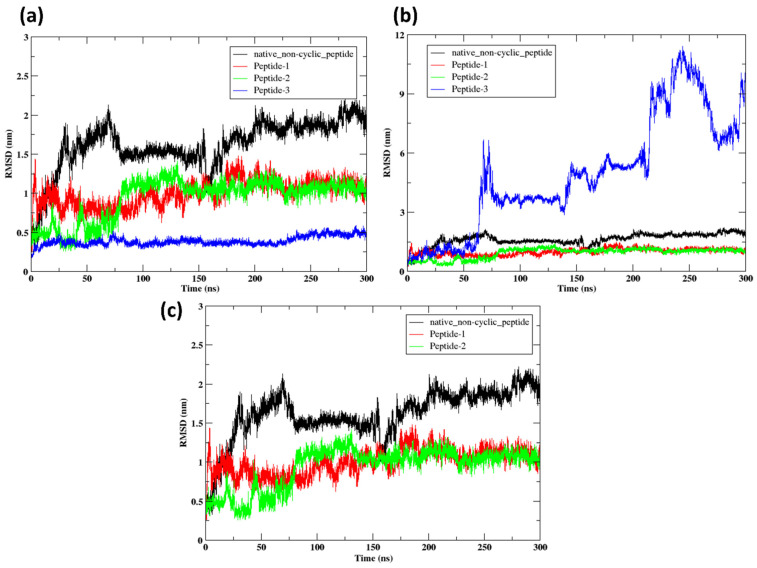
RMSD Analysis of Native Non-Cyclic and Modified Peptides: (**a**) RMSD of protein complex with native non-cyclic peptide and Peptides 1, 2, and 3 over 300 ns. (**b**) RMSD for native non-cyclic peptide and Peptides 1, 2, and 3 over 300 ns. (**c**) RMSD for native non-cyclic peptide, Peptide 1, and Peptide 2 showing relative stability.

Figure 9a shows the RMSD of the protein while interacting with the native non-cyclic peptide and the other three mutated peptides, 1, 2, and 3, during the 300 ns run. Here, the protein complex with Peptide 3 shows stable conformation at 0.1 nm throughout the run. While initially protein complex peptide 2 for the first 60 ns showed stable conformation at 0.5 nm, later it fluctuated and reached 1.2 nm and showed stability from 60 ns to 120 ns. Then, it fluctuated slightly downward and showed stability from 150 ns to 300 ns at 0.8 nm. Moreover, the protein complex with Peptide 1 fluctuated between 1 and 1.2 nm and showed stable confirmation. Additionally, the protein complex with the native non-cyclic peptide continuously fluctuated from 0.5 nm to 1.8 nm for the first 20 ns, then deviated from 1.5 nm to 2 nm, then deviated downwards and showed stable confirmation from 60 ns to 150 ns. Later, it highly deviated downwards at 1 nm. Subsequently, it deviates upwards at 1.8 nm and fluctuates between 2 nm and 2.2 nm. Overall, it showed after the mutation. Peptide 3 shows the most significant improvement in stability, followed by Peptide 2 and Peptide 1. The native non-cyclic peptide is the least stable, exhibiting the highest structural deviations over time.

Figure 9b shows the RMSD of the peptides. Here, Peptide 3 showed various fluctuations, and after 200 ns, it moved out of the system. Peptide 2 exhibits a consistently steady root mean square deviation (RMSD) ranging from 0.5 to 1.0 nm during the simulation, suggesting that it maintains a stable structure with minimal deviation from its initial conformation. Peptide 1 exhibits comparable stability to Peptide 2, as indicated by RMSD values ranging from 0.5 to 1.5 nm. Although there are slight variations, the general pattern indicates a consistent conformation throughout the simulation. In addition, the native non-cyclic peptide exhibits higher RMSD values than Peptide 1 and Peptide 2. Nevertheless, it remains relatively stable in comparison to Peptide 3. The RMSD values vary between 1.0 and 2.5 nm, which suggests moderate differences in structure. Overall, the analysis indicates that Peptide 2 and Peptide 1 are potentially more suitable options for maintaining structural integrity in the simulated conditions.

Figure 9c shows the RMSD of Peptide 1, Peptide 2, and native non-cyclic peptide. The native non-cyclic peptide exhibits RMSD ranging from 1.0 to 2.5 nm, suggesting considerable variations in its structure. Initially, there is a sharp increase in RMSD up to approximately 2.0 nm, following that it reaches an almost stable state but still exhibits variations, suggesting the presence of conformational flexibility or instability. Following an initial increase, the RMSD stabilizes within the range of 0.5 to 1.5 nm. This suggests that Peptide 1 consistently maintains a stable conformation with little deviations from its original structure. Peptide 2 exhibits the highest level of stability compared to the other two peptides, as seen by its constantly low RMSD values ranging from 0.5 to 1.0 nm. Peptide 2 exhibits minimal variations, but overall, it maintains its structural integrity effectively during the 300 ns simulation. Overall, Peptide 2 showed good stability with the lowest RMSD value.

#### 2.9.2. RMSF

The RMSF (Root Mean Square Fluctuation) analysis of the protein residues, when bound to various peptides (native non-cyclic peptide, Peptide 1, Peptide 2), provides valuable insights into the protein’s stability and flexibility. The RMSF values of the native non-cyclic peptide (black line) are consistently higher throughout the sequence, with significant maxima near the end, around residues 100, and 250, reaching up to 0.5 nm. This suggests that the protein is moderately flexible and less stable when bound to the native non-cyclic peptide. Alternatively, Peptide 1 (red line) induces lower RMSF values, which implies increased stability and reduced flexibility, particularly in the vicinity of residues 100, 200, and the end. The fluctuations in this region are of lower amplitude than those of the native peptide. The RMSF values of Peptide 2 (green line) are marginally higher than those of Peptide 1 but lower than those of the native non-cyclic peptide, suggesting moderate flexibility and stability. The most stable binding is provided by Peptide 1, as evidenced by the lowest RMSF values, according to comparative analysis. Peptide 2 offers reasonable stability with a slightly higher degree of flexibility. The RMSF values of the native non-cyclic peptide indicate that it is the least stable and the most flexible. This analysis emphasizes the stabilizing influence of Peptide 1 on the protein structure, thereby emphasizing its potential as the most effective peptide among the analyzed.

#### 2.9.3. SASA

**Figure 10:**
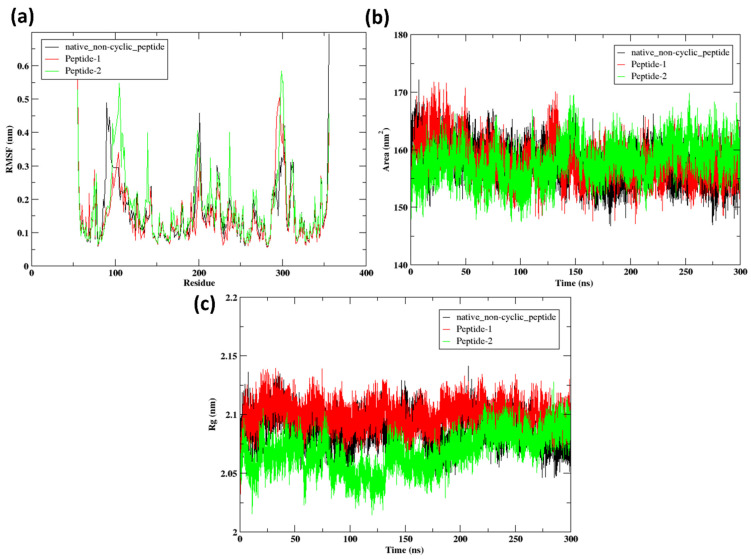
(**a**) RMSF of the protein when bound to peptides, (**b**) SASA of the protein when bound to the peptides, (**c**) Rg of the peptide.

Figure 10b shows the SASA of the peptides. The SASA values for the native non-cyclic peptide are approximately 150 to 170 nm^2^. This indicates a certain degree of structural flexibility, as evidenced by the consistent fluctuations around these values. However, the solvent exposure remains relatively consistent throughout the simulation. Peptide 1 exhibits SASA values that are within the range of approximately 150 to 175 nm^2^. The SASA values exhibit fluctuations that resemble those of the native peptide, however, with a little elevated peak. Peptide 1 exhibits a relatively larger surface area that is accessible to the solvent, suggesting a moderate level of stability with periodic variations in its form. Peptide 2 exhibits Solvent Accessible Surface Area (SASA) values ranging from approximately 140 to 170 nm^2^. Significant fluctuations are observed throughout the simulation, indicative of dynamic changes in solvent exposure. Notably, the overall SASA values of Peptide 2 are marginally lower than those of Peptide 1, implying a more compact conformation with reduced solvent accessibility. Overall, this indicates that Peptide 2 is the most stable and compact option, with low fluctuations in solvent exposure. Therefore, it is an improved option for applications that require stable structures.

#### 2.9.4. Radius of Gyration

Figure 10c shows the radius of gyration of peptides 1, 2, and the native non-cyclic peptide. In this context, The Rg values for the native non-cyclic peptide range between approximately 2.05 and 2.15 nm. The numbers exhibit fluctuations, suggesting that the native peptide maintains a consistent degree of compactness while maintaining structural flexibility over the simulation. The Rg values of Peptide 1 range from approximately 2.10 to 2.20 nm. The Rg values for Peptide 1 exhibit higher values compared to the native non-cyclic peptide, indicating that Peptide 1 possesses a slightly less condensed structure. The Rg values of Peptide 2 range from about 2.00 to 2.10 nm. The Rg values for Peptide 2 exhibit consistently lower and more stable values in comparison to both the native non-cyclic peptide and Peptide 1. This suggests that Peptide 2 maintains a highly compact structure with little variations during the simulation. Overall, it suggested that peptide 2 maintains the lowest Rg value indicating the most compact and stable structure among the three peptides.

It was identified that structural flexibility plays a crucial role in the inhibitory efficacy of peptides. The non-cyclic peptides demonstrated increased flexibility compared to the original cyclic S2Li5 peptide, which might contribute to their enhanced binding affinities and inhibitory activities. Specifically, the flexibility allows for better adaptation to the enzyme’s active site, optimizing interactions and leading to higher inhibition. In comparison, the cyclic S2Li5, with its constrained structure, may have limited conformational adaptability, potentially resulting in lower inhibition. The root-mean-square fluctuation (RMSF) values indicated that the non-cyclic peptides exhibited greater mobility in certain regions, allowing for more effective engagement with the active site residues of the deacetylase enzyme. Root-mean-square deviation (RMSD) analysis was used to compare the stability and conformational changes of the peptides during the MD simulations. The RMSD values of the non-cyclic peptides were higher than those of the cyclic S2Li5, indicating greater structural fluctuations and flexibility. This increased flexibility allows the non-cyclic peptides to adapt more effectively to the enzyme’s active site, optimizing interactions that enhance inhibition.

In contrast, the cyclic S2Li5 peptide exhibited lower RMSD values, suggesting a more rigid and stable structure. While this rigidity contributes to a well-defined conformation, it limits the peptide’s ability to adjust and form optimal interactions with the enzyme’s active site, potentially resulting in lower inhibition efficiency. Furthermore, the increased RMSD observed in non-cyclic peptides correlates with their higher binding affinities, as the flexibility enables them to explore a broader conformational space and stabilize more favorable interactions with the enzyme. This adaptability is crucial for enhancing binding and inhibitory activity.

### 2.10. Hydrogen Bonds

**Figure 11:**
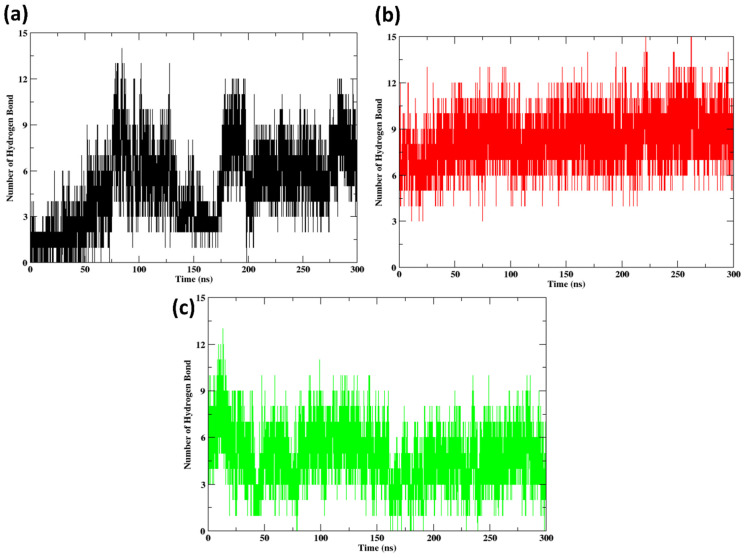
Hydrogen bonds formed between protein and the peptides for (**a**) Native non-cyclic peptide, (**b**) Peptide 1, (**c**) Peptide 2.

Hydrogen bonds between the protein and peptide at 300 ns are shown in Figure 11. It can be observed in Figure 11a that there are more fluctuations; initially, the number of hydrogen bonds is very low, i.e., 3–5, but later it changes to 10–12 at around 100 ns of the simulation. Then, later still, it starts showing a decreased number of hydrogen bonds in the range of 2–4. Gradually, the hydrogen bonds start to increase to 12 at around 200 ns of the simulation. In the case of Figure 11b, the hydrogen bonds show a stable state without further fluctuations compared to Figure 11a. The hydrogen bonds are in a range between 3 and 13 throughout the simulation, and there are no fluctuations shown. Similarly, in the case of peptide 2 (Figure 11c), the number of hydrogen bonds decreases slightly but remains within a narrower range of approximately 3 to 9. These differences imply varying environmental conditions or interaction strengths across the simulations; from the plot, we can identify that Figure 11a is less stable and that Figure 11b is more stable.

### 2.11. PCA and FEL

**Figure 12:**
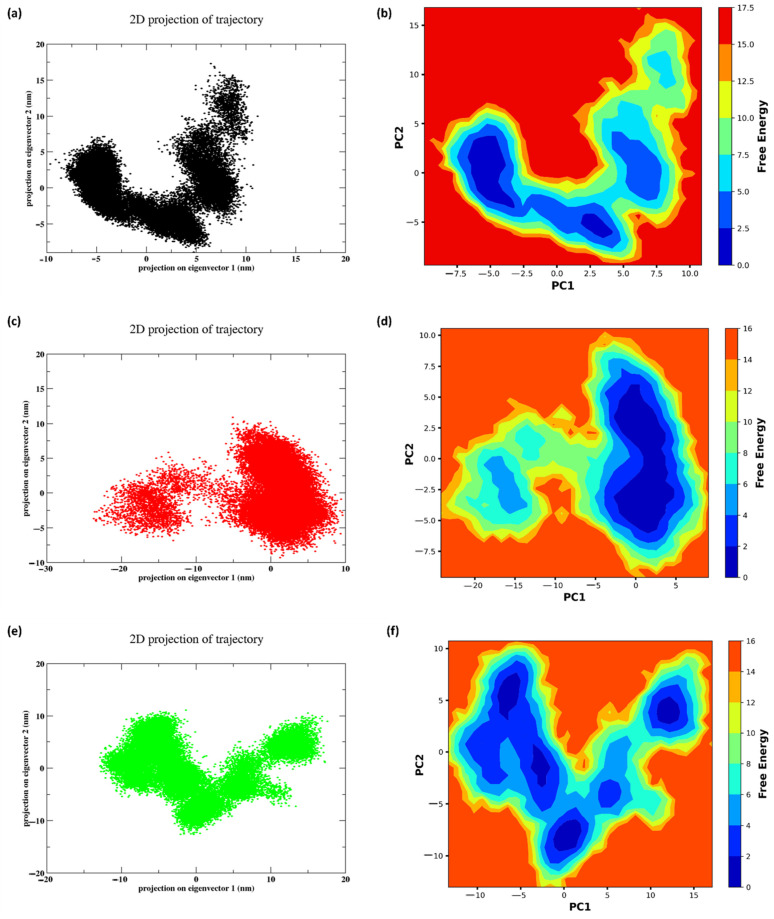
PCA projections and Free energy landscape of peptide bound to ligands and control during the 300 ns simulation: (**a**), (**b**) Native non-cyclic peptide (**c**), (**d**) Peptide 1 (**e**), (**f**) Peptide 2.

The Principal Component Analysis (PCA) is a scatter plot representation of the trajectory produced by the Molecular Dynamics (MD) simulation. Figure 12a features a U-shaped pattern, indicating two clusters connected by a smooth transition path, suggesting the presence of intermediate states by the native non-cyclic peptide during the MD run, showing its transition from the initial state to the final state. This implies moderate conformational changes, likely involving binding pocket dynamics and varying interaction strengths between compact and extended conformations. Similarly, in Figure 12c, peptide 1 forms two distinct clusters with some overlap, indicating distinct conformational states that may reflect specific binding poses or allosteric effects. The larger spread along the PC1 axis suggests significant structural changes or ligand binding modes, while the separation of clusters implies higher energy barriers between states, reflecting rigid conformational selectivity. In addition, in Figure 12e, peptide 2 presents a butterfly-shaped distribution with central convergence, indicating that the system frequently samples intermediate states, suggesting flexible binding dynamics and conformational adaptability. This pattern is indicative of a system that is energetically favorably exploring multiple conformations, possibly involving allosteric sites or multiple binding pathways.

Figure 12 shows the free energy landscape (FEL) and PCA of the peptides. In Figure 12b, the free energy landscape exhibits multiple distinct basins, with the regions of lowest free energy (dark blue) representing the most thermodynamically stable conformations. The basins are divided by areas of elevated free energy (yellow to red), indicating substantial energy barriers between distinct conformational states. This suggests that the native non-cyclic peptide exists in several stable structural states, with noticeable transitions occurring between them. Similarly, the landscape of peptide 1 formed several free energy basins, which are more interconnected than those of the original peptide. The existence of clearly defined basins characterized by low levels of free energy (shown by the dark blue color) signifies the presence of stable conformations. On the other hand, the connecting regions, which exhibit higher energy levels ranging from yellow to red, signify the presence of moderate energy barriers. In addition, peptide 2 formed multiple basins; however, the basins are less defined and more interconnected, with lower energy barriers (lighter shades of blue) between states, suggesting greater conformational flexibility. Overall, Peptide 2 exhibits a broader range of possible shapes with low energy barriers, resulting in more flexibility and a greater number of stable configurations.

### 2.12. MM/GBSA

**Figure 13:**
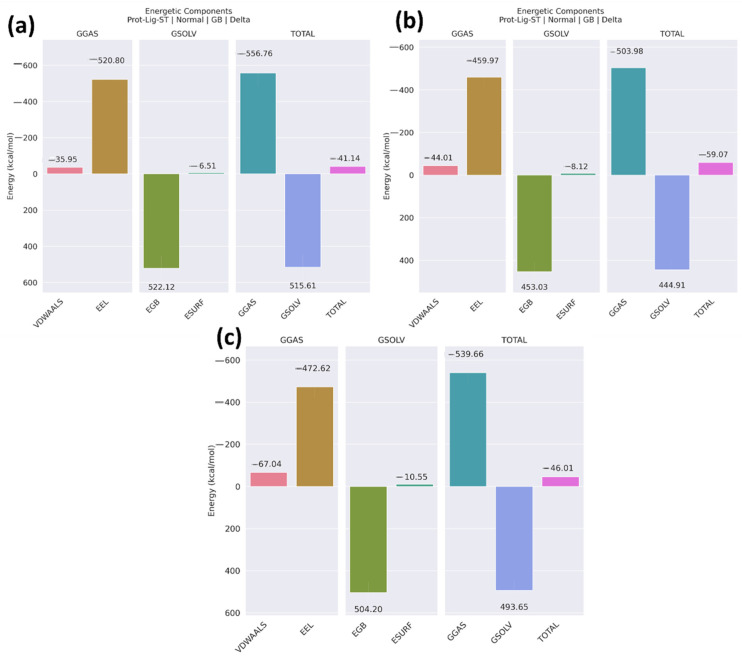
Binding free energy of the complexes of the protein and the peptides: (**a**) native non-cyclic peptide, (**b**) Peptide 1, (**c**) Peptide 2.

The binding free energy of the protein–ligand complex was measured, including different energetic components. Here, in Figure 13a, the total binding free energy of the native non-cyclic peptide complex was −41.14 kcal/mol ± 7.43, with the combination of GGAS and GSOLV. Figure 13b shows the total binding free energy of the peptide 1 complex as −59.07 kcal/mol ± 6.33, and Figure 13c shows the total binding energy of the peptide 2 complex as −46.01 kcal/mol ± 7.15. The error bar is also shown in Table 3. The van der Waals interactions are most favorable in peptide 2, suggesting robust hydrophobic interactions, while electrostatic energy (EEL) is most significant in the non-cyclic peptide (−520.80 kcal/mol ± 68.37). The non-polar solvation energy (ESURF) is highest in peptide 2 (−10.55 kcal/mol ± 0.77), indicating potential hydrophobic surface interactions. Overall, peptide 1’s combination of favorable van der Waals and solvation energies, along with relatively balanced electrostatic and polar contributions, makes it the most potent candidate, suggesting higher affinity and stability in the binding environment. This suggests that the peptide 1 complex shows a strong binding affinity with the protein molecule.

## 3. Methodology

### 3.1. Protein Structure

The 3D protein structure of sirtuin 2 (SIRT2) was retrieved from the Protein Data Bank (PDB) database with the PDB ID: 4L3O [20]. A peptide structure was bound to SIRT2 in the PDB structure. This ε-trifluoroacetyllysine-containing macrocyclic peptide can inhibit SIRT2 activity more potently than most other known inhibitors [19]. The crystal structure of human SIRT2 in complex with a macrocyclic peptide inhibitor, S2iL5, at 2.5 Å resolution was retrieved. S2iL5 contains ε-trifluoroacetyllysine and is part of a series of macrocyclic peptides that exhibit potent inhibitory activity against SIRT2. S2iL5 binds to the active site of SIRT2, inducing significant conformational changes, including an open-to-closed domain movement and a helix-to-coil transition in a specific region of the enzyme. This dynamic structural change is crucial for its potent inhibitory effect. The structure revealed that S2iL5 binds to the active site of SIRT2 through extensive interactions. The polymer sequence of S2iL5 is ACY-TYR-HIS-THR-TYR-HIS-VAL-FAK-ARG-ARG-THR-ASN-TYR-TYR-CYS-NH2, as shown in Figure 1.

Further, PEP-FOLD3 [21], a web-based tool for peptide structure prediction, was used to model the modified non-cyclic peptide. The top model generated by PEP-FOLD3 was selected for further analysis, ensuring the best structural representation of the modified peptide.

### 3.2. Native-Protein Peptide Docking

The target protein’s binding residues were found using the PyMOL program [22]. The binding residues were selected within a 6 Å radius of the ligand in the crystal structure. The H-Dock server [23], a novel web server designed for molecular docking, particularly for protein–protein and protein–DNA/RNA interactions. It uses a hybrid docking algorithm that combines template-based modeling with free docking, which delivers accurate protein–peptide docking predictions, to target dock the newly modified peptide. In total, 100 models were extracted from the docking procedure for further investigation.

### 3.3. MMGBSA

The energy minimization of protein–peptide was performed using the GROMACS 2022.4 package [24]. CHARMM36 [25] was employed to establish the molecular topology and force field parameters, which were assigned to both proteins and peptides. Later, the generation of the force-field parameters and topologies of the compounds and the control inhibitor, the CGenFF [26] server, was used. The Ewald Particle Mesh method was utilized in order to calculate the electrostatic force [27]. The system was solvated in the cubic box with the TIP3P water solvent model [28]. Na+ and Cl^−^ ions were subsequently introduced to perform the neutralization. Further, to eliminate steric conflicts, the system underwent 50,000 minimization steps utilizing the steepest descent (SD) method. Subsequently, the LINCS algorithm [29] was implemented to restrict the bonds, thus attaining system stability. Further, the MM/GBSA (Molecular Mechanics Generalized Born Surface Area) technique was used in this study [30,31]. The GROMACS tool gmx_MM/PBSA was used to calculate the binding free energy of the complexes until energy minimization. The equation involved in calculating binding free energy is described below:

(1) $$∆G=G_{complex\;\;}-[G_{receptor}+G_{ligand}]$$

(2) $${\Delta G}_{binding}=\Delta H-T\Delta S$$

(3) $$\Delta H=\Delta G_{GAS}+\Delta G_{SOLV}$$

(4) $$\Delta G_{GAS}=\Delta E_{EL}+\Delta E_{VDWAALS}$$

(5) $$\Delta G_{SOLV}=\Delta E_{GB}+\Delta E_{SURF}$$

(6) $$\Delta E_{SURF}=\gamma .SASA$$

In Equation (1), the variables *G_complex_*, *G_receptor_*, and *G_ligand_* represent the total free energies of the protein–peptide complex, the free enzyme, and the peptide in the solvent, respectively. The remaining Equations in (2)–(6) illustrated the alterations in gas-phase energy ΔG_GAS_, the solvation free energy change ΔG_SOLV_, the conformational entropy change −TΔS, and the enthalpy change ΔH. The investigation incorporated the solvent-accessible surface area (SASA) and the solvent’s surface tension (γ), both of which were displayed graphically. Changes in electrostatic and van der Waals energies were denoted by ΔE_VDWAALS_ and ΔE_EL_, respectively. Furthermore, as indicated in the research, the polar and nonpolar solvation energy changes were accurately represented by ΔE_GB_ and ΔE_SURF_. The model with the highest free binding energy was selected for molecular dynamics simulation.

### 3.4. Molecular Dynamics (MD) Simulation (100 ns)

The best model of the modified non-cyclic peptide and the known cyclic peptide complexed with SIRT2 was used for MD simulation of 100 ns. The method for energy minimization is given in Section 3.3. Later, the temperature of the entire system was increased to 310 K with a timestep of 2 fs throughout a 100 ps simulation in the NVT ensemble. The coordinates of the structure were stored at 10 ps intervals for the duration of the production run, which spanned 300 ns. In addition, the velocity scaling [32] method was employed as a temperature coupling in order to maintain stimulation at a consistent temperature. The Parrinello–Rahman [33] pressure coupling method was subsequently implemented to maintain constant pressure during the manufacturing process.

### 3.5. Mutation and QSAR

Further, mutation of the original peptide sequence was performed by targeting mutations at different positions. Using Python’s itertools.product, all possible single-residue mutations were generated using the 20 standard amino acids at the designated sites, yielding distinct peptide variations. These variations were generated by combining all possible amino acid combinations and inserting them into the original sequence. The dataset for training was downloaded from the PDBbind database (http://pdbbind.org.cn/ accessed on 25 January 2024) [34], and the protein and ligand sequences were encoded using multiple techniques before being split into training, validation, and testing sets. The quantitative structure–activity relationship (QSAR) approach has yielded precise and flexible findings in the realm of drug discovery. Since the traditional machine learning approach was employed in QSAR, the rise of big data and deep learning technologies has purposefully enhanced the processing of unstructured data and unlocked QSAR’s potential [35]. Here, Python, DeepPurpose [36], Matplotlib [37], NumPy [38], and Scikit-learn [39] were the primary tools used within a Jupyter Notebook environment. Later, for QSAR, three regression models were used, random forest regressor, Ridge regression, XGBoost, and gradient boosting regression model, which were trained and optimized with hyperparameter tuning. The best (hyperparameter) settings for every model must be identified in order to evaluate a model’s prediction potential with less bias. For machine learning algorithms to operate at their best, their hyperparameters must be adjusted [40]. Here, hypermeter tuning via Optuna [41] is carried out for the minimization of the root mean square error (RMSE). Later, an isolation forest was employed to detect and remove outliers, enhancing the robustness of the models. Subsequently, the QSAR model was built after compound properties were calculated using the RDkit programs [42]. Additionally, the models’ coefficients of determination (R^2^) were calculated in order to validate the trained models, and the model with the greatest value was chosen to be employed in the screening procedure. The model is deemed to have a good fit since the R2 value indicates that there is a substantial correlation between the predicted values and the actual values. The performances of the models were based on the R^2^ scores and RMSE, and the final model was saved using Pickle. In order to predict the activity, the data were sourced and processed, which included a target protein sequence and corresponding mutated peptides. These sequences were encoded using the Conjoint Triad method [43] and they are labeled for prediction. Lastly, 70% of the compounds were used in the training of the model, while the remaining 30% were used as test compounds. The isolation forest was employed to detect and remove the outliers, which helped to enhance the data quality. The predictions were made on the cleaned dataset, and the results were visualized for the evaluation of the model’s performance. The peptides that showed greater predictive activity compared to the non-cyclic control peptide were taken further for clustering.

### 3.6. Clustering

Clustering was performed after calculating the hydrophobicity values and molecular weights for each peptide using predefined scales. Subsequently, the data were normalized using standard scaling. Later, Principal Component Analysis (PCA) was performed to reduce the dimensionality of the feature space to two components. After PCA, the k-means function of the cluster module was utilized for clustering in the sklearn package of Python [39]. The matplotlib module in Python [44] was used to generate all of the plots. Following the clustering process, each cluster’s centroid—which represented the cluster as a whole—was retrieved. For each centroid, i.e., for 3 clusters, the closest peptides are identified based on their Euclidean distances. The three closest peptides to each centroid are selected for further analysis.

### 3.7. Peptide Modeling and Docking

The three closest peptides were used for modeling, and PEPFOLD.3 [21] was used for the modeling from which top models were selected for docking purposes. The method for docking is similar to the method given in Section 3.2. Later, the MD of all the top models was run till energy minimization and binding free energy using MMGBSA was calculated with the same method described in Section 3.3. The model that had the highest binding free energy was selected for MD analysis.

### 3.8. Molecular Dynamics Simulation (300 ns)

The detailed method of MD simulation is given in Section 3.3 and Section 3.4.

### 3.9. PCA and FEL

Principal component analysis (PCA) was performed on protein–ligand complexes using GROMACS’s default configurations. In order to prepare the trajectory to undergo principal component analysis, it was subjected to rigorous preparation that involved the elimination of periodic circumstances. Further, with the g‘mx covar’ tool included in GROMACS, the covariance matrix was calculated in order to calculate the better trajectory [45,46]. It is the covariance matrix that is responsible for characterizing the link between the atomic variation in the protein–ligand complex. The eigenvalues and eigenvectors of the covariance matrix were determined by employing the gmx anaeig function in the computation process. The PC coordinates for each frame were calculated utilizing the g‘mx anaproj’ GROMACS program so that the trajectory could be observed on the PCs.

A more comprehensive understanding of various processes, such as biomolecule identification, aggregation, and folding, can be attained through the investigation of the dynamics of biological systems [47]. The analysis of the system’s equilibrium state, as represented by the Free Energy Landscape (FEL) minima, and its non-equilibrium condition, as marked by the FEL barriers, can accomplish this purpose. In order to determine the FEL, the following equation was applied:

(7) $$∆G\left(X\right)=-kBTln\;P(X)$$

The ∆*G* represents Gibbs free energy, the Boltzmann constant is denoted by *kB*, the absolute temperature is denoted by *T*, *X* denotes the reaction coordinate, and *P*(*X*) denotes the probability distribution of the system along the reaction coordinate.

### 3.10. MM/GBSA

The detailed method of binding free energy is given in Section 3.2.

## 4. Conclusions

SIRT2, an NAD+-dependent deacetylase, plays a critical role in the regulation of aging, DNA repair, and cell-cycle progression. Abnormal SIRT2 activity is associated with diseases such as Parkinson’s, cancer, and metabolic disorders, making it an important focus for therapeutic discovery. Peptide based inhibitors have potential benefits in terms of selectivity and bioavailability. Thus, this study transformed a cyclic peptide inhibitor of SIRT2 into a non-cyclic form by removing the modified residue, FAK (LYS + CF_3_CO^−^). The tweaked peptide exhibited enhanced binding affinity in comparison to the already-known cyclic inhibitor S2iL5. The 3D structures of the peptides reveal distinct conformations that highlight their structural diversity and potential biological roles. The cyclic peptide exhibits a compact loop and beta-sheet-like features, suggesting a stable structure potentially suited for specific interactions. The alpha-helical peptides show varying degrees of helix formation, which is indicative of their flexibility and potential for diverse functional interactions. The presence of cysteine residues in some peptides suggests the formation of disulfide bridges, which can enhance stability and influence their biological activity. These structural insights provide valuable information for understanding the peptides’ potential applications in drug development and molecular interactions. The cyclic peptide S2iL5, with the sequence ACY-TYR-HIS-THR-TYR-HIS-VAL-FAK-ARG-ARG-THR-ASN-TYR-TYR-CYS-NH2, exhibited a binding free energy of −49.44 kcal/mol. Upon conversion to a non-cyclic form, the new sequence demonstrated an improved binding free energy of −50.66 kcal/mol. Through additional modification and screening, three peptides were discovered to be particularly promising. Among these, Peptide 1 and Peptide 2 demonstrated the most favorable binding free energy, with a value of −59.07 kcal/mol and −46.01 kcal/mol, respectively. The significant energy differences in van der Waals (vdW), electrostatic (EEL), and generalized Born electrostatic solvation (EGB) components among the peptides highlight the complexity of their interactions. Larger vdW differences suggest variations in surface complementarity, while substantial EEL differences indicate changes in electrostatic interactions due to specific mutations. Variations in EGB reflect different solvation effects. These energy component discrepancies emphasize the need to consider multiple factors in evaluating binding affinities and designing effective peptide inhibitors. Molecular dynamics analyses revealed that Peptide 1 and Peptide 2 not only maintained significant stability but also displayed structural flexibility, which is advantageous for effective inhibition. While Peptide 1 had a higher binding affinity than the original cyclic S2iL5, Peptide 2’s binding affinity was slightly lower, yet both non-cyclic peptides demonstrated potential enhancements in peptide-based inhibition over the original cyclic form. The results emphasize the potential of peptide-based inhibitors to improve the strength of binding and effectiveness, indicating that they could serve as viable alternatives to small molecules for targeting SIRT2. However, additional experimental studies are necessary to confirm these computational forecasts and evaluate the therapeutic capabilities of the discovered peptides.

## Figures and Tables

**Table 1:** R^2^ or the coefficient of determination of the models for the four models Random Forest, Ridge, GradientBoostingRegressor, and XGBRegressor.

Model	R^2^ on Training Set	R^2^ on Test Set
Random Forest	0.934	0.672
Ridge	0.952	−1.482
GradientBoostingRegressor	0.887	0.647
XGBRegressor	0.995	0.696

**Table 2:** ADME prediction of the peptides using ProtParam tool.

Parameter	Peptide 1	Peptide 2	Peptide 3
Molecular Weight	1707.88	1869.15	1650.83
Theoretical pI	9.63	9.11	9.63
Total Negatively Charged Residues (Asp + Glu)	0	1	0
Total Positively Charged Residues (Arg + Lys)	3	3	3
Total Number of Atoms	230	252	223
Aliphatic Index	20.71	20.71	20.71
Grand Average of Hydropathicity (GRAVY)	−1.579	−1.229	−1.357

**Table 3:** Comparative MMGBSA Results for Non-cyclic Peptide, Peptide 1, and Peptide 2.

Compounds	VDWAALS	EEL	EGB	ESURF	GGAS	GSOLV	Total
Non-cyclic peptide	−35.95 ± 7.36	−520.80 ± 68.37	522.12 ± 71.38	−6.51 ± 0.86	−556.76 ± 71.39	515.61 ± 76.78	−41.14 ± 7.43
Peptide 1	−44.01 ± 5.65	−459.97 ± 45.95	453.03 ± 45.51	−8.12 ± 45.51	−503.98 ± 46.37	444.91 ± 45.29	−59.07 ± 6.33
Peptide 2	−67.04 ± 5.42	−472.62 ± 54.15	504.20 ± 50.26	−10.55 ± 0.77	−539.66 ± 54.83	493.65 ± 49.87	−46.01 ± 7.15

## Data Availability

Data are contained within the article.

## Supplementary Materials

The following supporting information can be downloaded at: https://www.mdpi.com/article/10.3390/ph17091120/s1, Figure S1: Interaction plot of cyclic peptide S2iL5 with the protein SIRT2; Table S1:Binding free energy of the 100 models of the modified cyclic peptide and the protein SIRT2 using MM/GBSA; Table S2: Silhouette scores for different number of clusters.
