# Supplementary material for: Computational Design and Optimization of Peptide Inhibitors for SIRT2
Source: Pharmaceuticals (Basel). 2024 Aug 24;17(9):1120. doi: 10.3390/ph17091120 (PMC11435109; doi:10.3390/ph17091120)
Supplement: Supplementary file 1 [file pharmaceuticals-17-01120-s001.zip › pharmaceuticals-3114256-supplementary.pdf]

Supplementary

Table S1. Binding free energy of the 100 models of the modified cyclic peptide and the protein SIRT2 using MM/GBSA

Model	Energy (kcal/mol)	Model	Energy (kcal/mol)	Model	Energy (kcal/mol)	Model	Energy (kcal/mol)	Model	Energy (kcal/mol)
54	-37.19	39	-13.34	64	-7.39	53	-1.77	77	5.34
44	-26.34	24	-12.52	89	-7.11	80	-1.47	43	5.96
18	-26	69	-11.59	37	-7.01	35	-1.45	36	6.6
45	-24.58	26	-11.44	23	-6.32	90	-0.89	32	7.26
82	-22.99	92	-11.33	12	-6.13	84	-0.56	72	7.27
17	-22.58	66	-11.24	67	-6.01	95	-0.09	81	7.38
5	-22.45	13	-10.85	55	-5.97	27	-0.07	52	7.91
28	-22.45	6	-10.43	59	-5.76	73	0.12	21	8.22
56	-22.1	29	-9.81	70	-5.5	57	0.31	63	8.78
10	-21.81	49	-9.61	100	-5.04	1	0.37	30	9.42
99	-21.58	88	-9.44	97	-4.68	98	0.41	76	9.68
14	-21.48	61	-9.37	11	-4.08	34	2.14	2	11.19
20	-19.81	58	-9.34	78	-3.95	51	2.53	93	13.06
68	-18.39	50	-8.79	9	-3.79	75	2.92	74	13.54
79	-18.2	19	-8.77	41	-3.71	62	2.94	87	15.61
4	-17.58	38	-8.69	60	-3.71	91	3.44	86	15.73
65	-17.06	33	-8.59	47	-3.65	3	3.58	8	21.78
42	-17.03	71	-8.3	48	-3.15	31	4.37	40	22.27
83	-14.96	94	-7.57	25	-2.79	7	4.57	16	23.64
85	-14.91	22	-7.5	46	-1.94	96	4.85	15	24.71

Table S2. Silhouette scores for different number of clusters

Number of clusters	Silhouette score
**2**	0.3403
**3**	0.3888
**4**	0.3387
**5**	0.3381
**6**	0.3319
**7**	0.3310

**8**	0.3333
**9**	0.3323
**10**	0.3414

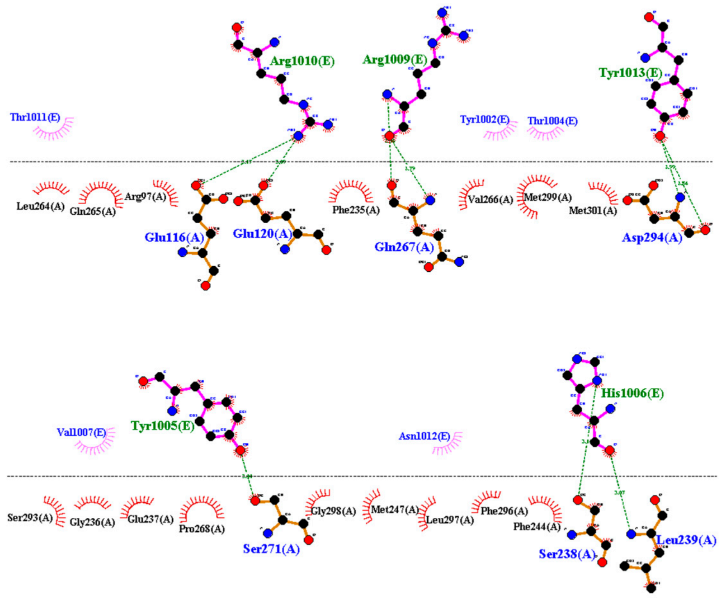

Figure S1. Interaction plot of cyclic peptide S2iL5 with the protein SIRT2
